# Deepfakes Generation and Detection: A Short Survey

**DOI:** 10.3390/jimaging9010018

**Published:** 2023-01-13

**Authors:** Zahid Akhtar

**Affiliations:** Department of Network and Computer Security, State University of New York (SUNY) Polytechnic Institute, Utica, NY 13502, USA; akhtarz@sunypoly.edu; Tel.: +1-315-792-7238

**Keywords:** DeepFakes, digital face manipulations, digital forensics, fake news, multimedia manipulations, generative AI, deepfake generation, deepfake detection, deep learning, face recognition, misinformation, disinformation face morphing attack, biometrics, fake news, information authenticity

## Abstract

Advancements in deep learning techniques and the availability of free, large databases have made it possible, even for non-technical people, to either manipulate or generate realistic facial samples for both benign and malicious purposes. DeepFakes refer to face multimedia content, which has been digitally altered or synthetically created using deep neural networks. The paper first outlines the readily available face editing apps and the vulnerability (or performance degradation) of face recognition systems under various face manipulations. Next, this survey presents an overview of the techniques and works that have been carried out in recent years for deepfake and face manipulations. Especially, four kinds of deepfake or face manipulations are reviewed, i.e., identity swap, face reenactment, attribute manipulation, and entire face synthesis. For each category, deepfake or face manipulation generation methods as well as those manipulation detection methods are detailed. Despite significant progress based on traditional and advanced computer vision, artificial intelligence, and physics, there is still a huge arms race surging up between attackers/offenders/adversaries (i.e., DeepFake generation methods) and defenders (i.e., DeepFake detection methods). Thus, open challenges and potential research directions are also discussed. This paper is expected to aid the readers in comprehending deepfake generation and detection mechanisms, together with open issues and future directions.

## 1. Introduction

It is estimated that 1.8 billion images and videos per day are uploaded to online services, including social and professional networking sites [1]. However, approximately 40% to 50% of these images and videos appear to be manipulated [2] for benign reasons (e.g., images retouched for magazine covers) or adversarial purposes (e.g., propaganda or misinformation campaigns). In particular, human face image/video manipulation is a serious issue menacing the integrity of information on the Internet and face recognition systems since faces play a central role in human interactions and biometrics-based person identification. Therefore, plausible manipulations in face samples can critically subvert trust in digital communications and security applications (e.g., law enforcement).

**Figure 1:**
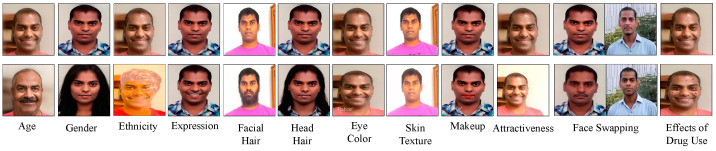
Examples of different face manipulations: original samples (first row) and manipulated samples (second row).

DeepFakes refer to multimedia content that has been digitally altered or synthetically created using deep learning models [3]. Deepfakes are the results of face swapping, enactment/animation of facial expressions, and/or digitally generated audio or non-existing human faces. In contrast, face manipulation involves modifying facial attributes such as age, gender, ethnicity, morphing, attractiveness, skin color or texture, hair color, style or length, eyeglass, makeup, mustache, emotion, beard, pose, gaze, mouth open or closed, eye color, injury and effects of drug use [4,5], and adding imperceptible perturbations (i.e., adversarial examples), as shown in Figure 1. The readily-available face editing apps (e.g., FaceApp [6], ZAO [7], Face Swap Live [8], Deepfake web [9], AgingBooth [10], PotraitPro Studio [11], Reface [12], Audacity [13], Soundforge [14], Adobe Photoshop [15]), and Deep Neural network (DNN) source codes [16,17] have enabled even non-experts and non-technical people to create sophisticated deepfakes and altered face samples, which are difficult to be detected by human examiners and current image/video analysis forensics tools.

Deepfakes are expected to advance present disinformation and misinformation sources to the next level, which could be exploited by trolls, bots, conspiracy theorists, hyperpartisan media, and foreign governments; thus, deepfakes could be fake news 2.0. Deepfakes can be used for productive applications such as realistic dubbing of foreign video films [18] or historical figure reanimation for education [19]. Deepfakes can also be used for destructive applications such as the use of fake pornographic videos to damage a person’s reputation or to blackmail them [20], manipulating elections [21], creating warmongering situations [22], generating political or religious unrest via fake speeches [23], causing chaos in financial markets [24], or identity theft [25]. It is easy to notice that the number of malevolent exploitations of deepfakes chiefly dominates the benevolent ones. In fact, not only have recent advances made creating a deepfake with just a still image [26], but also deepfakes are successfully being misused by cybercriminals in the real world. For instance, an audio deepfake was utilized to scam a CEO out of $243,000 [27]. The issue of deepfakes and face manipulations is getting compounded as they can negatively affect the automated face recognition system (AFRS). For instance, studies have shown that AFRS’s error rates can reach up to 95% under deepfakes [28], 50–99% under morphing [29], 17.08% under makeup manipulation [30], 17.05–99.77% under partial face tampering [31], 40–74% under digital beautification [32], 93.82% under adversarial examples [33], and 67% under GANs generated synthetic samples [34]. Similarly, automated speaker verification’s accuracy drops to 40% from 98% under adversarial examples [35].

There exist many deepfake and face manipulation detection methods. However, a systematic analysis shows that the majority of them have low generalization capability, i.e., their performances drop drastically when they encounter a novel deepfake/manipulation type that was not used during the training stage, as also demonstrated in [36,37,38,39,40]. Also, prior studies considered deepfake detection a reactive defense mechanism and not as a battle between the attackers (i.e., deepfake generation methods) and the defenders (i.e., deepfake detection methods) [41,42,43]. Therefore, there is a crucial gap between academic deepfake solutions and real-world scenarios or requirements. For instance, the foregoing works are usually lagging in the robustness of the systems against adversarial attacks [44], decision explainability [45], and real-time mobile deepfake detection [46].

The study of deepfake generation and detection, in recent years, is gathering much more momentum in the computer vision and machine learning community. There exist some review papers on this topic (e.g., [5,24,47,48]), but they are focused mainly on deepfake or synthetic samples using generative adversarial networks. Moreover, most survey articles (e.g., [4,49,50]) were mainly written from an academic point of view and not from a practical development point of view. Also, they did not cover the advent of very recent face manipulation methods and new deepfake generation and detection techniques. Thus, this paper provides a concise but comprehensive overview from both theoretical and practical points of view to furnish the reader with an intellectual grasp as well as to facilitate the progression of novel and more resilient techniques. For example, publicly available apps, codes, or software information can be easily accessed or downloaded for further development and use. All in all, this paper presents an overview of current deepfake and face manipulation techniques by covering four kinds of deepfake or face manipulation. The four main types of manipulation are identity swap, face reenactment, attribute manipulation, and entire face synthesis, where every category manipulation generation and such manipulation detection methods are summarized. Furthermore, open challenges and potential future directions (e.g., robust deepfake detection systems against adversarial attacks using multistream and filtering schemes) that need to be addressed in this evolving field of deepfakes are highlighted. The main objectives of this article are to complement earlier survey papers with recent advancements, to impart to the reader a deeper understanding of the deepfake creation and detection domain, and to use this article as ground truth to develop novel algorithms for deepfake and face manipulation generation and detection systems. 

The rest of the article is organized as follows. Section 2 presents deepfake and face manipulation generation as well as detection techniques. In Section 3, the open issues and potential future directions of deepfake generation and detection are discussed. The conclusions are described in Section 4.

## 2. Deepfake Generation and Detection

**Figure 2:**
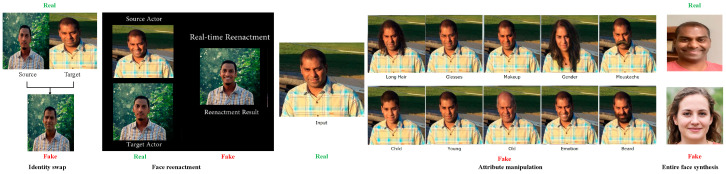
Real and fake examples of each deepfake/face manipulation group. The fake sample in “Entire face synthesis” group is obtained from the method in [81].

We can broadly define deepfake as “believable audio-, visual- or multimedia generated by deep neural networks”. Deepfake/face manipulation can be categorized into four main groups: identity swap, face reenactment, attribute manipulation, and entire face synthesis [47], as shown in Figure 2. Several works have been conducted on different types of deepfake/face manipulation generation and detection. However, in the following subsections, we have included representative studies based on their novelty, foundational idea, and/or performance. Also, studies have been incorporated to represent the most up-to-date research works depicting the state-of-the-art in deepfake generation and detection. 

### 2.1. Identity Swap

Here, an overview of existing identity swap or face swap (i.e., replacing a person’s face with another person’s face) generation and detection methods is presented.

#### 2.1.1. Identity Swap Generation

This consists of replacing the face of a person in the target image/video with the face of another person in the source image/video [51]. For example, Korshunova et al. [52] developed a face-swapping method using Convolutional Neural Networks (CNNs). While Nirkin et al. [53] proposed a technique using a standard fully convolutional network in unconstrained settings. Mahajan et al. [54] presented a face swap procedure for privacy protection. Wang et al. [55] presented a real-time face-swapping method. Natsume et al. [56] proposed a region-separative generative adversarial network (RSGAN) for face swapping and editing. Other interesting face swamping methods can be seen in [28,57,58,59,60,61]. 

#### 2.1.2. Identity Swap Detection

Ample studies have been conducted on identity swap deepfake detection. For instance, Koopman et al. [62] analyzed photo response non-uniformity (PRNU) for detection. Also, warping artifacts [63], eye blinking [64], optical flow with CNNs [65], heart rate [66], image quality [28], local image textures [37], long short-term memory (LSTM) and recurrent neural network (RNN) [67], multi-LSTM and blockchain [68], clustering [69], context [70], compression artifacts [71], metric learning [72], CNN ensemble [73], Identity-aware [74], transformers [75], audio-visual dissonance [76], and multi-attentional [77] features were used. Very few works have been focused on deepfake detection method’s explainability (e.g., [78]) and generalization capability (e.g., work of Bekci et al. in [38] and Aneja et al. [79] work using zero-shot learning). Recently, S. Liu et al. [80] proposed a block shuffling learning method to detect deepfakes, where the image is divided into blocks, and using random shuffling where intra-block and inter-block-based features are extracted. 

### 2.2. Face Reenactment

Here, an overview of prior face reenactment (i.e., changing the facial expression of the individual) generation and detection techniques is provided.

#### 2.2.1. Face Reenactment Generation

This consists of replacing the facial expression of a person in the target image/video with the facial expression of another person in the source image/video [47]. It is also known as expression swap or puppet master. For instance, Thies et al. [82] developed real-time face reenactment RGB video streams. Whereas encoder-decoder, RNN, unified landmark converter with geometry-aware generator, GANs, and task-agnostic GANs-based schemes were designed by Kim et al. [83], Nirkin et al. [84], Zhang et al. [85], Doukas et al. [86], and Cao et al. [87], respectively. 

#### 2.2.2. Face Reenactment Detection 

Face reenactment detection methods were designed by Cozzolino et al. [88] using CNNs; Matern et al. [89] using visual features with logistic regression and MLP; Rossler et al. [90] using mesoscopic, steganalysis, and CNN features; Sabir et al. [91] using RNN; Amerini et al. [65] using Optical Flow + CNNs; Kumar et al. [92] using multistream CNNs; and Wang et al. [93] using 3DCNN. In contrast, Zhao et al. [94] designed a spatiotemporal network, which can utilize complementary global and local information. In particular, the framework uses a spatial module for the global information, and the local information module extracts features from patches selected by attention layers. 

### 2.3. Attribute Manipulation

Here, an overview of existing attribute manipulation or face retouching, or face editing (i.e., altering certain face attributes such as skin tone, age, and gender) generation and detection techniques is presented.

#### 2.3.1. Attribute Manipulation Generation 

This consists of modifying some facial attributes, e.g., color of hair/skin, gender, age, adding glasses [95,96,97]. It is also known as face editing or face retouching. Xiao et al. [98] presented a multi-attribute manipulation GANs-based system. Moreover, spatial attention in GANs [99], variational autoencoder (VAE) + GANs [100], multi-domain GANs [101], geometry-aware GANs [102], mask-guided GANs [103], 3D face morphable model [104], and GIMP animation [105] based methods have been designed. 

#### 2.3.2. Attribute Manipulation Detection

In [36], authors studied the efficacy of different deep learning models’ efficacy for attribute manipulation detection. The Deep Boltzmann machine by Bharati et al. [106], CNN by Dang et al. [107], LBP + landmarks + CNNs by Rathgeb et al. [108], adaptive manipulation traces by Guo et al. [109], encoder-decoder by Mazaheri et al. [110], facial boundary features by Kim et al. [111], and PRNU by Scherhag et al. [112] were exploited.

### 2.4. Entire Face Synthesis

Here, an overview of prior entire face synthesis (i.e., creating non-existent face samples) generation and detection techniques is provided.

#### 2.4.1. Entire Face Synthesis Generation 

This consists of generating entire non-existent face images [113,114,115]. Berthelot et al. [116] developed boundary equilibrium GANs to create synthetic faces. Similarly, various approaches have been devised, e.g., coupled GANs [117], invertible convolution [118], U-Net [119], from speech to face GANs [120], high-resolution deep convolutional GANs [121], interactive anycost GANs [122], and structured disentanglement framework for face generation and editing [123].

#### 2.4.2. Entire Face Synthesis Detection 

Many studies have also focused on entire face synthesis detection. For example, McCloskey et al. [124] presented a color cues-based system. While GAN fingerprint + CNNs [125], PRNU [126], co-occurrence matrices [127], neuron behaviors [128], incremental learning + CNNs [129], and self-attention mechanism [130] were also utilized. Table 1 presents a summary of deepfake and face manipulation generation and detection techniques. Guo et al. [131] showed that GANs-generated faces could be detected by analyzing the irregular pupil shapes, which may be caused by the lack of physiological constraints in the GANs models. 

## 3. Open Issues and Research Directions

Although great efforts have been made in devising deepfake generation and detection, there are several issues yet to be addressed successfully. In the following, some of them are discussed.

### 3.1. Generalization Capability

It is easy to notice in the literature that most of the existing deepfake detection frameworks’ performances decrease remarkably when tested under deepfakes, manipulations, or databases that were not used for the training. Thus, detecting unknown novel deepfakes or deepfake generation tools is yet a big challenge. The generalization capability of deepfake detectors is vital for dependable precision and public trust in the information being shared online. Some preliminary generalization solutions have been proposed, but their ability to tackle novel emerging deepfakes is still an open issue. 

### 3.2. Explainability of Deepfake Detectors 

There is a lack of work on the deepfake detection framework’s interpretability and dependability. Most deep-learning-based deepfake or face manipulation detection methods in the literature usually do not explain the reason behind the final detection outcome. It is mainly due to deep learning techniques being the black box in nature. Current deepfake or face manipulation detectors only give a label, confidence percentage, or fakeness probability score but not the insight description of results. Such a description would be useful to know why the detector made a certain decision. Also, deepfake or face manipulation (e.g., applying digital makeup) can be performed either for benign or malicious intentions. Nonetheless, present deepfake or face manipulation detection techniques cannot distinguish the intent. For deepfake detection framework’s interpretability and dependability, various advanced combinations of techniques such as fuzzy inference systems [187], layer-wise relevance propagation [188], and the Neural Additive Model [189] could be helpful.

### 3.3. Next-Generation Deepfake and Face Manipulation Generators

Improved deepfake and face manipulation generation techniques will help develop more advanced and generalized deepfake detection methods. Some of the shortcomings of current datasets and generation methods are the lack of ultra-high-resolution samples (e.g., existing methods are usually generating 1014 × 1024 resolution samples, which is not sufficient for the next generation of deepfakes), limited face attribution manipulations (i.e., face attribute manipulation types are dependent on the training set, thereby manipulation characteristics and attributes are limited, and novel attributes cannot be generated), video continuity problem (i.e., the deepfake/face manipulation, especially identity swap, techniques neglects the continuation of video frames as well as physiological signals), and no obvious deepfake/face manipulations (i.e., present databases are not composed of obvious fake samples such as a human face with three eyes).

### 3.4. Vulnerability to Adversarial Attacks

Recent studies have shown that deep learning-based deepfake and face manipulation detection methods are vulnerable to adversarial examples [44]. Though current detectors are capable of handling several degradations (e.g., compression and noise), their accuracy goes to extremely low levels under adversarial attacks. Thus, next-generation techniques should be not only able to tackle deepfakes but also adversarial examples. To this aim, developing various multistream and filtering schemes could be effective.

### 3.5. Mobile Deepfake Detector

The neural networks-based deepfake detection methods, which are capable of attaining remarkable accuracy, are mostly unsuited for mobile platforms/applications owing to the huge number of parameters and computational cost. Compressed, yet effective, deep learning-based detection systems, which could be used on mobile and wearable devices, will greatly help counteract deepfakes and fake news.

### 3.6. Lack of Large-Scale ML-Generated Databases

Most studies on AI-synthesized face sample detection compiled their own database with various GANs. Thereby, different published studies have different performances on GANs samples, because the quality of GANs-generated samples varies and are mostly unknown. Several public GANs-generated fake face sample databases should be produced to help the advancement of this demanding research field.

### 3.7. Reproducible Research

In machine learning and the deepfake research community, the reproducible results trend should be urged by furnishing the public with large datasets with larger human scores/reasons, experimental setups, and open-source tools/codes. It will surely aid in outlining the true progress in the field and avoid overestimation of the performances by the developed methods.

## 4. Conclusions

AI-synthesized or digitally manipulated face samples, commonly known as DeepFakes, are a significant challenge threatening the dependability of face recognition systems and the integrity of information on the Internet. This paper provides a survey on recent advances in deepfake and facial manipulation generation and detection. Despite noticeable progress, there are several issues remaining to be resolved to attain highly effective and generalized generation and defense techniques. Thus, this article discussed some of the open challenges and research opportunities. The field of deepfakes still has to go a long way for dependable deepfake and face manipulation detection frameworks, which will need interdisciplinary research efforts in various domains, such as machine learning, computer vision, human vision, psychophysiology, etc. All in all, this survey may be utilized as a ground truth for developing novel AI-based algorithms for deepfake generation and detection. Also, it is hoped that this survey paper will motivate budding scientists, practitioners, researchers, and engineers to consider deepfakes as their domain of study.

## Figures and Tables

**Table 1:** Representative works on deepfake and face manipulation generation and detection techniques. SWR = successful swap rate; MS-SSIM = multi-scale structural similarity; Acc = accuracy; LL = Logloss; AUC = area under the curve; CL = contextual loss; RMSE = root mean square error; AU = Facial action unit; CSIM = Cosine Similarity between IMage embeddings; EER = Equal error rate; FID = Frechet inception distance; AP = Average Precision; KID = kernel inception distance; PSNR = Peak Signal-to-Noise Ratio.

Study	Approach	Dataset	Performance	Source Code	Year
**Deepfake Generation**					
Wang et al. [55]	Real-time face swapping using CANDIDE-3	COFW [132], 300W [133], LFW [134]	SWR = 87.9%.	×	2018
Natsume et al. [56]	Face swapping and editing using RSGAN	CelebA [135]	MS-SSIM = 0.087	×	2018
Chen et al. [61]	High fidelity encoder-decoder	VGGFace2 [136]	Qualitative Analysis	https://github.com/neuralchen/SimSwap (accessed on 4 January 2023)	2021
Xu et al. [137]	Lightweight Identity-aware Dynamic Network	VGGFace2 [136] FaceForensics++ [90]	FID = 6.79%	https://github.com/Seanseattle/MobileFaceSwap (accessed on 4 January 2023)	2022
Shu et al. [138]	Portrait, identity, and pose encoders with generator and feature pyramid network	VoxCeleb2 [139]	PSNR = 33.26	https://github.com/jmliu88/heser (accessed on 4 January 2023)	2022
**Deepfake Detection**					
Afcha et al. [140]	CNNs	FaceForensics++ [90]	Acc = 98.40%	https://github.com/DariusAf/MesoNet (accessed on 4 January 2023)	2018
Zhao et al. [77]	Multi-attentional	FaceForensics++ [90] DFDC [3]	Acc = 97.60% LL = 0.1679	https://github.com/yoctta/multiple-attention (accessed on 4 January 2023)	2021
Miao et al. [141]	Transformers via bag-of-feature for generalization	FaceForensics++ [90], Celeb-DF [142], DeeperForensics-1.0 [143]	Acc = 87.86% AUC = 82.52% Acc = 97.01%	×	2021
Prajapati et al. [144]	Perceptual Image Assessment + GANs	DFDC [3]	AUC = 95% Acc = 91%	https://github.com/pratikpv/mri_gan_deepfake (accessed on 4 January 2023)	2022
Wang et al. [75]	Multi-modal Multi-scale Transformer (M2TR)	FaceForensics++ [90]	Acc = 97.93%	https://github.com/wangjk666/M2TR-Multi-modal-Multi-scale-Transformers-for-Deepfake-Detection (accessed on 4 January 2023)	2022
**Reenactment Generation**					
Zhang et al. [145]	Decoder + warping	CelebA-HQ [146] FFHQ [147] RAF-DB [148]	AU = 75.1% AU = 70.9% AU = 71.1%	https://github.com/bj80heyue/One_Shot_Face_Reenactment (accessed on 4 January 2023)	2019
Ngo et al. [149]	Encoder-decoder	300VW [150]	CL= 1.46	×	2020
Tripathy et al. [151]	Facial attribute controllable GANs	FaceForensics++ [90]	CSIM = 0.747	×	2021
Bounareli et al. [152]	3D shape model	VoxCeleb [153]	FID = 0.66	×	2022
Agarwal et al. [154]	Audio-Visual Face Reenactment GAN	VoxCeleb [153]	FID = 9.05	https://github.com/mdv3101/AVFR-Gan/ (accessed on 4 January 2023)	2023
**Reenactment Detection**					
Nguyen et al. [155]	Autoencoder	FaceForensics++ [90]	EER = 7.07%	https://github.com/nii-yamagishilab/ClassNSeg (accessed on 4 January 2023)	2019
Dang et al. [156]	CNNs + Attention mechanism	FaceForensics++ [90]	AUC = 99.4% EER = 3.4%	https://github.com/Jstehouwer/FFD_CVPR2020 (accessed on 4 January 2023)	2020
Kim et al. [157]	Knowledge Distillation	FaceForensics++ [90]	Acc = 86.97%	×	2021
Yu et al. [158]	U-Net Structure	FaceForensics++ [90]	Acc = 97.26%	×	2022
Wu et al. [159]	Multistream Vision Transformer Network	FaceForensics++ [90]	Acc = 94.46%	×	2022
**Attribute Manipulation Generation**					
Lample et al. [160]	Encoder-decoder	CelebA [135]	RMSE = 0.0009	https://github.com/facebookresearch/FaderNetworks (accessed on 4 January 2023)	2018
Liu et al. [161]	Selective transfer GANs	CelebA [135]	Acc = 70.80%	https://github.com/csmliu/STGAN (accessed on 4 January 2023)	2019
Kim et al. [162]	Real-time style map GANs	CelebA-HQ [146] AFHQ [163]	FID = 4.03 FID = 6.71	https://github.com/naver-ai/StyleMapGAN (accessed on 4 January 2023)	2021
Huang et al. [164]	Multi-head encoder and decoder	CelebA-HQ [146] StyleMapGAN [162]	MSE = 0.023 FID = 7.550	×	2022
Sun et al. [165]	3D-aware generator with two decoupled latent codes	FFHQ [147]	FID = 28.2	https://github.com/MrTornado24/FENeRF (accessed on 4 January 2023)	2022
**Attribute Manipulation Detection**					
Wang et al. [166]	CNNs	Own dataset	Acc = 90.0%	https://github.com/peterwang512/FALdetector (accessed on 4 January 2023)	2019
Du et al. [167]	DFT + CNNs	Deepfake-in-the-wild [168] Celeb-DF [142] DFDC [3]	Acc = 78.00% Acc = 96.00% Acc = 81.00%	×	2020
Akhtar et al. [36]	DNNs	Own dataset	Acc = 99.31	×	2021
Rathgeb et al. [169]	Human majority voting	FERET [170]	CCR = 62.8%	×	2022
Guo et al. [171]	Gradient operator convolutional network with tensor pre-processing and manipulation trace attention module	FaceForensics++ [90]	Acc = 94.86%	https://github.com/EricGzq/GocNet-pytorch (accessed on 4 January 2023)	2023
**Entire face synthesis generation**					
Li et al. [172]	Conditional self-attention GANs	CelebA-HQ [146]	KID = 0.62	https://github.com/LiYuhangUSTC/Lines2Face (accessed on 4 January 2023)	2019
Karras et al. [81]	StyleGAN	FFHQ [147]	FID = 3.31	https://github.com/NVlabs/stylegan2 (accessed on 4 January 2023)	2020
Xia et al. [173]	Textual descriptions GANs	CelebA-HQ [146]	FID = 106.37	https://github.com/IIGROUP/TediGAN (accessed on 4 January 2023)	2021
Song et al. [174]	Text-to-speech system	LibriTTS dataset [175] AISHELL-3 [176]	FPS = 30.3	×	2022
Li et al. [177]	StyleT2I: High-Fidelity Text-to-Image Synthesis	CelebA-HQ [146]	FID = 18.02	https://github.com/zhihengli-UR/StyleT2I (accessed on 4 January 2023)	2022
**Entire face synthesis detection**					
Wang et al. [178]	CNNs	StyleGAN2 [81] ProGAN [146]	AP = 99.10% AP = 100%	https://github.com/peterwang512/CNNDetection (accessed on 4 January 2023)	2020
Pu et al. [179]	Incremental clustering	PGGAN [146]	F1 Score = 99.09%	https://github.com/jmpu/NoiseScope (accessed on 4 January 2023)	2020
Yousaf et al. [180]	Two-Stream CNNs	StarGAN [101]	Acc = 96.32%	×	2021
Nowroozi et al. [181]	Cross-band and spatial co-occurrence matrix + CNNs	StyleGAN2 [81] VIPPrint [182]	Acc = 93.80% Acc = 92.56%	×	2022
Boyd et al. [183]	Human-annotated saliency maps into a deep learning loss function	StyleGAN2 [81], ProGAN [146], StyleGAN [147], StyleGAN2-ADA [184], StyleGAN3 [185], StarGANv2 [163], SREFI [186]	AUC = 0.633	https://github.com/BoydAidan/CYBORG-Loss (accessed on 4 January 2023)	2023

## Data Availability

Data sharing not applicable.

## References

[1] https://theconversation.com/3-2-billion-images-and-720-000-hours-of-video-are-shared-online-daily-can-you-sort-real-from-fake-148630.

[2] https://www.nbcnews.com/business/consumer/so-it-s-fine-if-you-edit-your-selfies-not-n766186.

[3] Dolhansky B., Bitton J., Pflaum B., Lu J., Howes R., Wang M., Ferrer C. (2020). The deepfake detection challenge dataset. arXiv.

[4] Akhtar Z., Dasgupta D., Banerjee B. Face Authenticity: An Overview of Face Manipulation Generation, Detection and Recognition. Proceedings of the International Conference on Communication and Information Processing (ICCIP).

[5] Mirsky Y., Lee W. (2021). The creation and detection of deepfakes: A survey. ACM Comput. Surv..

[6] FaceApp Technology Limited. https://www.faceapp.com/.

[7] Laan Labs. http://faceswaplive.com/.

[8] Changsha Shenduronghe Network Technology Co., Ltd.. https://apps.apple.com/cn/app/id1465199127.

[9] DeepfakesWeb.com. https://deepfakesweb.com/.

[10] PiVi&Co. https://apps.apple.com/us/app/agingbooth/id35746779.

[11] Anthropics Technology Ltd.. https://www.anthropics.com/portraitpro/.

[12] Neocortext. https://hey.reface.ai/.

[13] The Audacity Team. https://www.audacityteam.org/.

[14] Magix Software GmbH. https://www.magix.com/us/music-editing/sound-forge/.

[15] Adobe. https://www.photoshop.com/en.

[16] Collins E., Bala R., Price B., Susstrunk S. Editing in style: Uncovering the local semantics of GANs. Proceedings of the IEEE/CVF Conference on Computer Vision and Pattern Recognition.

[17] He Z., Zuo W., Kan M., Shan S., Chen X. (2019). AttGAN: Facial attribute editing by only changing what you want. IEEE Trans. Image Process..

[18] Roettgers J. (2019). How AI Tech Is Changing Dubbing, Making Stars Like David Beckham Multilingual. https://variety.com/2019/biz/news/ai-dubbing-david-beckham-multilingual-1203309213/.

[19] Lee D. (2019). Deepfake Salvador Dali Takes Selfies with Museum Visitors, The Verge. https://www.theverge.com/2019/5/10/18540953/salvador-dali-lives-deepfake-museum.

[20] Güera D., Delp E.J. Deepfake Video Detection Using Recurrent Neural Networks. Proceedings of the 15th IEEE International Conference on Advanced Video and Signal Based Surveillance (AVSS).

[21] Diakopoulos N., Johnson D. (2021). Anticipating and addressing the ethical implications of deepfakes in the context of elections. New Media Soc..

[22] Pantserev K. (2020). The malicious use of AI-based deepfake technology as the new threat to psychological security and political stability. Cyber Defence in the Age of AI, Smart Societies and Augmented Humanity.

[23] Oliveira L. (2017). The current state of fake news. Procedia Comput. Sci..

[24] Zhou X., Zafarani R. (2020). A survey of fake news: Fundamental theories, detection methods, and opportunities. ACM Comput. Surv. (CSUR).

[25] Kietzmann J., Lee L., McCarthy I., Kietzmann T. (2020). Deepfakes: Trick or treat?. Bus. Horiz..

[26] Zakharov E., Shysheya A., Burkov E., Lempitsky V. Few-Shot Adversarial Learning of Realistic Neural Talking Head Models. Proceedings of the IEEE/CVF International Conference on Computer Vision (ICCV).

[27] Damiani J. (2019). A Voice Deepfake Was Used to Scam a CEO Out of $243,000. https://www.forbes.com/sites/jessedamiani/2019/09/03/a-voice-deepfake-was-used-to-scam-a-ceo-out-of-243000/?sh=173f55a52241.

[28] Korshunov P., Marcel S. Vulnerability assessment and detection of Deepfake videos. Proceedings of the International Conference on Biometrics (ICB).

[29] Scherhag U., Nautsch A., Rathgeb C., Gomez-Barrero M., Veldhuis R.N., Spreeuwers L., Schils M., Maltoni D., Grother P., Marcel S. Biometric Systems under Morphing Attacks: Assessment of Morphing Techniques and Vulnerability Reporting. Proceedings of the International Conference of the Biometrics Special Interest Group.

[30] Rathgeb C., Drozdowski P., Busch C. Detection of Makeup Presentation Attacks based on Deep Face Representations. Proceedings of the 25th International Conference on Pattern Recognition (ICPR).

[31] Majumdar P., Agarwal A., Singh R., Vatsa M. Evading Face Recognition via Partial Tampering of Faces. Proceedings of the IEEE/CVF Conference on Computer Vision and Pattern Recognition Workshops.

[32] Ferrara M., Franco A., Maltoni D., Sun Y. On the impact of alterations on face photo recognition accuracy. Proceedings of the International Conference on Image Analysis and Processing.

[33] Yang L., Song Q., Wu Y. (2021). Attacks on state-of-the-art face recognition using attentional adversarial attack generative network. Multimed. Tools Appl..

[34] Colbois L., Pereira T., Marcel S. (2021). On the use of automatically generated synthetic image datasets for benchmarking face recognition. arXiv.

[35] Huang C.-Y., Lin Y.Y., Lee H.-Y., Lee L.-S. Defending Your Voice: Adversarial Attack on Voice Conversion. Proceedings of the IEEE Spoken Language Technology Workshop (SLT).

[36] Akhtar Z., Mouree M.R., Dasgupta D. Utility of Deep Learning Features for Facial Attributes Manipulation Detection. Proceedings of the IEEE International Conference on Humanized Computing and Communication with Artificial Intelligence (HCCAI).

[37] Akhtar Z., Dasgupta D. A Comparative Evaluation of Local Feature Descriptors for DeepFakes Detection. Proceedings of the IEEE International Symposium on Technologies for Homeland Security (HST).

[38] Bekci B., Akhtar Z., Ekenel H.K. Cross-Dataset Face Manipulation Detection. Proceedings of the 28th Signal Processing and Communications Applications Conference (SIU).

[39] Khodabakhsh A., Akhtar Z. (2021). Unknown presentation attack detection against rational attackers. IET Biom..

[40] Yavuzkilic S., Sengur A., Aktar Z., Siddique K. (2021). Spotting DeepFakes and Face Manipulations by Fusing Features from Multi-Stream CNNs Models. Symmetry.

[41] Wang T., Cheng H., Chow K., Nie L. (2022). Deep convolutional pooling transformer for deepfake detection. arXiv.

[42] Kaddar B., Fezza S., Hamidouche W., Akhtar Z., Hadid A. HCiT: Deepfake Video Detection Using a Hybrid Model of CNN features and Vision Transformer. Proceedings of the 2021 IEEE Visual Communications and Image Processing (VCIP).

[43] Yavuzkiliç S., Akhtar Z., Sengür A., Siddique K. (2021). DeepFake Face Video Detection using Hybrid Deep Residual Networks and LSTM Architecture. AI and Deep Learning in Biometric Security: Trends, Potential and Challenges.

[44] Hussain S., Neekhara P., Jere M., Koushanfar F., McAuley J. Adversarial deepfakes: Evaluating vulnerability of deepfake detectors to adversarial examples. Proceedings of the IEEE/CVF Winter Conference on Applications of Computer Vision.

[45] Lim S.-Y., Chae D.-K., Lee S.-C. (2022). Detecting Deepfake Voice Using Explainable Deep Learning Techniques. Appl. Sci..

[46] Mehta V., Gupta P., Subramanian R., Dhall A. FakeBuster: A DeepFakes detection tool for video conferencing scenarios. Proceedings of the International Conference on Intelligent User Interfaces-Companion.

[47] Juefei-Xu F., Wang R., Huang Y., Guo Q., Ma L., Liu Y. (2022). Countering Malicious DeepFakes: Survey, Battleground, and Horizon. Int. J. Comput. Vis..

[48] Lu Z., Li Z., Cao J., He R., Sun Z. Recent progress of face image synthesis. Proceedings of the 4th IAPR Asian Conference on Pattern Recognition (ACPR).

[49] Zhang T. (2022). Deepfake generation and detection, a survey. Multimed. Tools Appl..

[50] Mustak M., Salminen J., Mäntymäki M., Rahman A., Dwivedi Y. (2023). Deepfakes: Deceptions, mitigations, and opportunities. J. Bus. Res..

[51] Tolosana R., Vera-Rodriguez R., Fierrez J., Morales A. (2020). Ortega-Garcia. Deepfakes and beyond: A survey of face manipulation and fake detection. Inf. Fusion.

[52] Korshunova I., Shi W., Dambre J., Theis L. Fast Face-Swap Using Convolutional Neural Networks. Proceedings of the IEEE International Conference on Computer Vision (ICCV).

[53] Nirkin Y., Masi I., Tuan A.T., Hassner T., Medioni G. On Face Segmentation, Face Swapping, and Face Perception. Proceedings of the 13th IEEE International Conference on Automatic Face & Gesture Recognition.

[54] Mahajan S., Chen L., Tsai T. SwapItUp: A Face Swap Application for Privacy Protection. Proceedings of the IEEE 31st International Conference on Advanced Information Networking and Applications (AINA).

[55] Wang H., Dongliang X., Wei L. Robust and Real-Time Face Swapping Based on Face Segmentation and CANDIDE-3. Proceedings of the PRICAI 2018: Trends in Artificial Intelligence.

[56] Natsume R., Yatagawa T., Morishima S. (2018). RSGAN: Face Swapping and Editing Using Face and Hair Representation in Latent Spaces. arXiv.

[57] Yan S., He S., Lei X., Ye G., Xie Z. Video face swap based on autoencoder generation network. Proceedings of the International Conference on Audio, Language and Image Processing (ICALIP).

[58] Zhou H., Liu Y., Liu Z., Luo P., Wang X. Talking face generation by adversarially disentangled audio-visual representation. Proceedings of the AAAI Conference on Artificial Intelligence.

[59] Li L., Bao J., Yang H., Chen D., Wen F. (2019). Faceshifter: Towards high fidelity and occlusion aware face swapping. arXiv.

[60] Li L., Bao J., Yang H., Chen D., Wen F. Advancing High Fidelity Identity Swapping for Forgery Detection. Proceedings of the IEEE/CVF Conference on Computer Vision and Pattern Recognition (CVPR).

[61] Chen R., Chen X., Ni B., Ge Y. SimSwap: An Efficient Framework For High Fidelity Face Swapping. Proceedings of the 28th ACM International Conference on Multimedia.

[62] Koopman M., Rodriguez A., Geradts Z. Detection of deepfake video manipulation. Proceedings of the 20th Irish Machine Vision and Image Processing Conference (IMVIP).

[63] Li Y., Lyu S. Exposing DeepFake Videos by Detecting Face Warping Artifacts. Proceedings of the IEEE Conference on Computer Vision and Pattern Recognition Workshops (CVPRW).

[64] Li Y., Chang M., Lyu S. (2018). In ictu oculi: Exposing ai generated fake face videos by detecting eye blinking. arXiv.

[65] Amerini I., Galteri L., Caldelli R., Del Bimbo A. Deepfake Video Detection through Optical Flow Based CNN. Proceedings of the IEEE/CVF International Conference on Computer Vision Workshop (ICCVW).

[66] Fernandes S., Raj S., Ortiz E., Vintila I., Salter M., Urosevic G., Jha S. Predicting Heart Rate Variations of Deepfake Videos using Neural ODE. Proceedings of the IEEE/CVF International Conference on Computer Vision Workshop (ICCVW).

[67] Tariq S., Lee S., Woo S. (2020). A Convolutional LSTM based Residual Network for Deepfake Video Detection. arXiv.

[68] Chan C.C.K., Kumar V., Delaney S., Gochoo M. Combating Deepfakes: Multi-LSTM and Blockchain as Proof of Authenticity for Digital Media. Proceedings of the IEEE/ITU International Conference on Artificial Intelligence for Good (AI4G).

[69] Zhu K., Wu B., Wang B. Deepfake Detection with Clustering-based Embedding Regularization. Proceedings of the IEEE Fifth International Conference on Data Science in Cyberspace (DSC).

[70] Nirkin Y., Wolf L., Keller Y., Hassner T. (2020). DeepFake detection based on the discrepancy between the face and its context. arXiv.

[71] Frick R.A., Zmudzinski S., Steinebach M. (2020). Detecting “DeepFakes” in H.264 Video Data Using Compression Ghost Artifacts. Electron. Imaging.

[72] Kumar A., Bhavsar A., Verma R. Detecting deepfakes with metric learning. Proceedings of the IEEE International Workshop on Biometrics and Forensics (IWBF).

[73] Bonettini N., Cannas E., Mandelli S., Bondi L., Bestagini P., Tubaro S. Video Face Manipulation Detection Through Ensemble of CNNs. Proceedings of the 25th International Conference on Pattern Recognition (ICPR).

[74] Cozzolino D., Rössler A., Thies J., Nießner M., Verdoliva L. (2020). ID-Reveal: Identity-aware DeepFake Video Detection. arXiv.

[75] Wang J., Wu Z., Ouyang W., Han X., Chen J., Jiang Y., Li S. M2TR: Multi-modal multi-scale transformers for deepfake detection. Proceedings of the International Conference on Multimedia Retrieval.

[76] Chugh K., Gupta P., Dhall A., Subramanian R. Not made for each other-Audio-Visual Dissonance-based Deepfake Detection and Localization. Proceedings of the 28th ACM International Conference on Multimedia.

[77] Zhao H., Zhou W., Chen D., Wei T., Zhang W., Yu N. Multi-attentional deepfake detection. Proceedings of the IEEE/CVF Conference on Computer Vision and Pattern Recognition.

[78] Trinh L., Tsang M., Rambhatla S., Liu Y. Interpretable and Trustworthy Deepfake Detection via Dynamic Prototypes. Proceedings of the IEEE/CVF Winter Conference on Applications of Computer Vision.

[79] Aneja S., Nießner M. (2020). Generalized Zero and Few-Shot Transfer for Facial Forgery Detection. arXiv.

[80] Liu S., Lian Z., Gu S., Xiao L. (2022). Block shuffling learning for Deepfake Detection. arXiv.

[81] Karras T., Laine S., Aittala M., Hellsten J., Lehtinen J., Aila T. Analyzing and improving the image quality of stylegan. Proceedings of the IEEE/CVF Conference on Computer Vision and Pattern Recognition.

[82] Thies J., Zollhofer M., Stamminger M., Theobalt C., Nießner M. Face2face: Real-time face capture and reenactment of RGB videos. Proceedings of the IEEE conference on computer vision and pattern recognition.

[83] Kim H., Garrido P., Tewari A., Xu W., Thies J., Niessner M., Pérez P., Richardt C., Zollhofer M., Theobalt C. (2018). Deep video portraits. ACM Trans. Graph. (TOG).

[84] Nirkin Y., Keller Y., Hassner T. FSGAN: Subject agnostic face swapping and reenactment. Proceedings of the IEEE/CVF International Conference on Computer Vision.

[85] Zhang J., Zeng X., Wang M., Pan Y., Liu L., Liu Y., Ding Y., Fan C. Freenet: Multi-identity face reenactment. Proceedings of the IEEE/CVF Conference on Computer Vision and Pattern Recognition.

[86] Doukas M., Koujan M., Sharmanska V., Roussos A., Zafeiriou S. (2021). Head2Head++: Deep Facial Attributes Re-Targeting. IEEE Trans. Biom. Behav. Identity Sci..

[87] Cao M., Huang H., Wang H., Wang X., Shen L., Wang S., Bao L., Li L., Luo J. (2020). Task-agnostic Temporally Consistent Facial Video Editing. arXiv.

[88] Cozzolino D., Thies J., Rossler A., Riess C., Niener M., Verdoliva L. (2018). Forensictransfer: Weakly-supervised domain adaptation for forgery detection. arXiv.

[89] Matern F., Riess C., Stamminger M. Exploiting Visual Artifacts to Expose DeepFakes and Face Manipulations. Proceedings of the IEEE Winter Applications of Computer Vision Workshops.

[90] Rossler A., Cozzolino D., Verdoliva L., Riess C., Thies J., Nießner M. Faceforensics++: Learning to detect manipulated facial images. Proceedings of the IEEE/CVF International Conference on Computer Vision.

[91] Sabir E., Cheng J., Jaiswal A., AbdAlmageed W., Masi I., Natarajan P. Recurrent Convolutional Strategies for Face Manipulation Detection in Videos. Proceedings of the IEEE/CVF Conference on Computer Vision and Pattern Recognition Workshops.

[92] Kumar P., Vatsa M., Singh R. Detecting face2face facial reenactment in videos. Proceedings of the IEEE/CVF Winter Conference on Applications of Computer Vision.

[93] Wang Y., Dantcheva A. A video is worth more than 1000 lies. Comparing 3DCNN approaches for detecting deepfakes. Proceedings of the IEEE International Conference on Automatic Face and Gesture Recognition (FG).

[94] Zhao X., Yu Y., Ni R., Zhao Y. Exploring Complementarity of Global and Local Spatiotemporal Information for Fake Face Video Detection. Proceedings of the 2022 IEEE International Conference on Acoustics, Speech and Signal Processing (ICASSP).

[95] Berthouzoz F., Li W., Dontcheva M., Agrawala M. (2011). A Framework for content-adaptive photo manipulation macros: Application to face, landscape, and global manipulations. ACM Trans. Graph..

[96] Lu J., Sunkavalli K., Carr N., Hadap S., Forsyth D. (2016). A visual representation for editing face images. arXiv.

[97] Ning X., Xu S., Nan F., Zeng Q., Wang C., Cai W., Jiang Y. (2022). Face editing based on facial recognition features. IEEE Trans. Cogn. Dev. Syst..

[98] Xiao T., Hong J., Ma J. Elegant: Exchanging latent encodings with gan for transferring multiple face attributes. Proceedings of the European Conference on Computer Vision (ECCV).

[99] Zhang G., Kan M., Shan S., Chen X. Generative adversarial network with spatial attention for face attribute editing. Proceedings of the European Conference on Computer Vision (ECCV).

[100] Sun R., Huang C., Zhu H., Ma L. (2021). Mask-aware photorealistic facial attribute manipulation. J. Comput. Visual Media.

[101] Choi Y., Choi M., Kim M., Ha J., Kim S., Choo J. StarGAN: Unified generative adversarial networks for multi-domain image-to-image translation. Proceedings of the IEEE Conference on Computer Vision and Pattern Recognition.

[102] Huang D., Tao X., Lu J., Do M.N. Geometry-Aware GAN for Face Attribute Transfer. Proceedings of the IEEE International Conference on Image Processing (ICIP).

[103] Wei Y., Gan Z., Li W., Lyu S., Chang M., Zhang L., Gao J., Zhang P. MagGAN: High-Resolution Face Attribute Editing with Mask-Guided Generative Adversarial Network. Proceedings of the Asian Conference on Computer Vision.

[104] Xu Z., Yu X., Hong Z., Zhu Z., Han J., Liu J., Ding E., Bai X. (2021). FaceController: Controllable Attribute Editing for Face in the Wild. arXiv.

[105] Ferrara M., Franco A., Maltoni D. The magic passport. Proceedings of the IEEE International Joint Conference on Biometrics.

[106] Bharati A., Singh R., Vatsa M., Bowyer K. (2016). Detecting facial retouching using supervised deep learning. IEEE Trans. Inf. Secur..

[107] Dang L.M., Hassan S.I., Im S., Moon H. (2019). Face image manipulation detection based on a convolutional neural network. Expert Syst. Appl..

[108] Rathgeb C., Satnoianu C.-I., Haryanto N.E., Bernardo K., Busch C. (2020). Differential Detection of Facial Retouching: A Multi-Biometric Approach. IEEE Access.

[109] Guo Z., Yang G., Chen J., Sun X. (2020). Fake face detection via adaptive residuals extraction network. arXiv.

[110] Mazaheri G., Roy-Chowdhury A. (2021). Detection and Localization of Facial Expression Manipulations. arXiv.

[111] Kim D., Kim D., Kim K. (2021). Facial Manipulation Detection Based on the Color Distribution Analysis in Edge Region. arXiv.

[112] Scherhag U., Debiasi L., Rathgeb C., Busch C., Uhl A. (2019). Detection of Face Morphing Attacks Based on PRNU Analysis. IEEE Trans. Biom. Behav. Identit-Sci..

[113] Zhao J., Mathieu M., LeCun Y. (2016). Energy-based generative adversarial network. arXiv.

[114] Kossaifi J., Tran L., Panagakis Y., Pantic M. Gagan: Geometry-aware generative adversarial networks. Proceedings of the IEEE Conference on Computer Vision and Pattern Recognition.

[115] Kaneko T., Hiramatsu K., Kashino K. Generative attribute controller with conditional filtered generative adversarial networks. Proceedings of the IEEE Conference on Computer Vision and Pattern Recognition.

[116] Berthelot D., Schumm T., Metz L. (2017). Began: Boundary equilibrium generative adversarial networks. arXiv.

[117] Liu M., Tuzel O. Coupled generative adversarial networks. Proceedings of the Advances in Neural Information Processing Systems 29 (NIPS 2016).

[118] Kingma D., Dhariwal P. (2018). Glow: Generative flow with invertible 1 × 1 convolutions. arXiv.

[119] Schonfeld E., Schiele B., Khoreva A. A u-net based discriminator for generative adversarial networks. Proceedings of the IEEE/CVF Conference on Computer Vision and Pattern Recognition.

[120] Choi H., Park C., Lee K. (2020). From inference to generation: End-to-end fully self-supervised generation of human face from speech. arXiv.

[121] Curtó J., Zarza I., De La Torre F., King I., Lyu M. (2017). High-resolution deep convolutional generative adversarial networks. arXiv.

[122] Lin J., Zhang R., Ganz F., Han S., Zhu J. Anycost gans for interactive image synthesis and editing. Proceedings of the IEEE/CVF Conference on Computer Vision and Pattern Recognition.

[123] Chen S., Liu F., Lai Y., Rosin P., Li C., Fu H., Gao L. (2021). DeepFaceEditing: Deep Face Generation and Editing with Disentangled Geometry and Appearance Control. arXiv.

[124] McCloskey S., Albright M. (2018). Detecting gan-generated imagery using color cues. arXiv.

[125] Yu N., Davis L., Fritz M. Attributing fake images to gans: Learning and analyzing gan fingerprints. Proceedings of the IEEE/CVF International Conference on Computer Vision.

[126] Marra F., Gragnaniello D., Verdoliva L., Poggi G. Do GANs leave artificial fingerprints?. Proceedings of the IEEE Conference on Multimedia Information Processing and Retrieval (MIPR).

[127] Nataraj L., Mohammed T.M., Manjunath B.S., Chandrasekaran S., Flenner A., Bappy J.H., Roy-Chowdhury A. (2019). Detecting GAN generated Fake Images using Co-occurrence Matrices. Electron. Imaging..

[128] Wang R., Juefei-Xu F., Ma L., Xie X., Huang Y., Wang J., Liu Y. (2019). Fakespotter: A simple yet robust baseline for spotting ai-synthesized fake faces. arXiv.

[129] Marra F., Saltori C., Boato G., Verdoliva L. Incremental learning for the detection and classification of gan-generated images. Proceedings of the IEEE International Workshop on Information Forensics and Security (WIFS).

[130] Li S., Dutta V., He X., Matsumaru T. (2022). Deep Learning Based One-Class Detection System for Fake Faces Generated by GAN Network. Sensors.

[131] Guo H., Hu S., Wang X., Chang M.C., Lyu S. Eyes Tell All: Irregular Pupil Shapes Reveal GAN-Generated Faces. Proceedings of the IEEE International Conference on Acoustics, Speech and Signal Processing (ICASSP).

[132] Burgos-Artizzu X., Perona P., Dollar P. Robust face landmark estimation under occlusion. Proceedings of the IEEE International Conference on Computer Vision.

[133] Sagonas C., Tzimiropoulos G., Zafeiriou S., Pantic M. 300 faces in-the-wild challenge: The first facial landmark localization challenge. Proceedings of the IEEE International Conference on Computer Vision Workshops.

[134] Learned-Miller E., Huang G., Chowdhury A., Li H., Hua G. (2016). Labeled Faces in the Wild: A Survey. Adv. Face Detect. Facial Image Anal..

[135] Liu Z., Luo P., Wang X., Tang X. Deep learning face attributes in the wild. Proceedings of the IEEE International Conference on Computer Vision (ICCV).

[136] Cao Q., Shen L., Xie W., Parkhi O.M., Zisserman A. VGGFace2: A Dataset for Recognising Faces across Pose and Age. Proceedings of the 13th IEEE International Conference on Automatic Face & Gesture Recognition (FG).

[137] Xu Z., Hong Z., Ding C., Zhu Z., Han J., Liu J., Ding E. (2022). MobileFaceSwap: A Lightweight Framework for Video Face Swapping. arXiv.

[138] Shu C., Wu H., Zhou H., Liu J., Hong Z., Ding C., Han J., Liu J., Ding E., Wang J. Few-Shot Head Swapping in the Wild. Proceedings of the IEEE/CVF Conference on Computer Vision and Pattern Recognition.

[139] Chung J.S., Nagrani A., Zisserman A. Voxceleb2: Deepspeaker recognition. Proceedings of the IEEE Conf. Conference of the International Speech Communication Association.

[140] Afchar D., Nozick V., Yamagishi J., Echizen I. Mesonet: A compact facial video forgery detection network. Proceedings of the IEEE International Workshop on Information Forensics and Security (WIFS).

[141] Miao C., Chu Q., Li W., Gong T., Zhuang W., Yu N. (2021). Towards Generalizable and Robust Face Manipulation Detection via Bag-of-local-feature. arXiv.

[142] Li Y., Yang X., Sun P., Qi H., Lyu S. Celeb-df: A large-scale challenging dataset for deepfake forensics. Proceedings of the IEEE/CVF Conference on Computer Vision and Pattern Recognition.

[143] Jiang L., Li R., Wu W., Qian C., Loy C. Deeperforensics-1.0: A large-scale dataset for real world face forgery detection. Proceedings of the IEEE/CVF Conference on Computer Vision and Pattern Recognition.

[144] Prajapati P., Pollett C. (2022). MRI-GAN: A Generalized Approach to Detect DeepFakes using Perceptual Image Assessment. arXiv.

[145] Zhang Y., Zhang S., He Y., Li C., Loy L.C.C., Liu Z. One-shot Face Reenactment. Proceedings of the British Machine Vision Conference (BMVC).

[146] Karras T., Aila T., Laine S., Lehtinen J. (2017). Progressive growing of gans for improved quality, stability, and variation. arXiv.

[147] Karras T., Laine S., Aila T. A style-based generator architecture for generative adversarial networks. Proceedings of the IEEE/CVF Conference on Computer Vision and Pattern Recognition.

[148] Li S., Deng W., Du J. Reliable Crowdsourcing and Deep Locality-Preserving Learning for Expression Recognition in the Wild. Proceedings of the IEEE Conference on Computer Vision and Pattern Recognition (CVPR).

[149] Ngo L., Karaoglu S., Gever T. Unified Application of Style Transfer for Face Swapping and Reenactment. Proceedings of the Asian Conference on Computer Vision.

[150] Shen J., Zafeiriou S., Chrysos G.G., Kossaifi J., Tzimiropoulos G., Pantic M. The first facial landmark tracking in-the-wild challenge: Benchmark and results. Proceedings of the IEEE International Conference on Computer Vision Workshops.

[151] Tripathy S., Kannala J., Rahtu E. FACEGAN: Facial Attribute Controllable rEenactment GAN. Proceedings of the IEEE/CVF Winter Conference on Applications of Computer Vision.

[152] Bounareli S., Argyriou V., Tzimiropoulos G. (2022). Finding Directions in GAN’s Latent Space for Neural Face Reenactment. arXiv.

[153] Nagrani A., Chung J.S., Zisserman A. Voxceleb: A large-scale speaker identification dataset. Proceedings of the INTERSPEECH.

[154] Agarwal M., Mukhopadhyay R., Namboodiri V., Jawahar C. Audio-visual face reenactment. Proceedings of the IEEE/CVF Winter Conference on Applications of Computer Vision.

[155] Nguyen H., Fang F., Yamagishi J., Echizen I. (2019). Multi-task Learning For Detecting and Segmenting Manipulated Facial Images and Videos. arXiv.

[156] Dang H., Liu F., Stehouwer J., Liu X., Jain A. On the Detection of Digital Face Manipulation. Proceedings of the IEEE/CVF Conference on Computer Vision and Pattern Recognition.

[157] Kim M., Tariq S., Woo S. FReTAL: Generalizing Deepfake Detection using Knowledge Distillation and Representation Learning. Proceedings of the IEEE/CVF Conference on Computer Vision and Pattern Recognition.

[158] Yu P., Fei J., Xia Z., Zhou Z., Weng J. (2022). Improving Generalization by Commonality Learning in Face Forgery Detection. IEEE Trans. Inf. Secur..

[159] Wu H., Wang P., Wang X., Xiang J., Gong R. GGViT:Multistream Vision Transformer Network in Face2Face Facial Reenactment Detection. Proceedings of the 2022 26th International Conference on Pattern Recognition (ICPR).

[160] Lample G., Zeghidour N., Usunier N., Bordes A., Denoyer L., Ranzato M. (2017). Fader networks: Manipulating images by sliding attributes. arXiv.

[161] Liu M., Ding Y., Xia M., Liu X., Ding E., Zuo W., Wen S. STGAN: A unified selective transfer network for arbitrary image attribute editing. Proceedings of the IEEE/CVF Conference on Computer Vision and Pattern Recognition.

[162] Kim H., Choi Y., Kim J., Yoo S., Uh Y. Exploiting Spatial Dimensions of Latent in GAN for Real-Time Image Editing. Proceedings of the IEEE/CVF Conference on Computer Vision and Pattern Recognition.

[163] Choi Y., Uh Y., Yoo J., Ha J. StarGAN v2: Diverse image synthesis for multiple domains. Proceedings of the IEEE/CVF Conference on Computer Vision and Pattern Recognition.

[164] Huang W., Tu S., Xu L. (2023). IA-FaceS: A Bidirectional Method for Semantic Face Editing. Neural Netw..

[165] Sun J., Wang X., Zhang Y., Li X., Zhang Q., Liu Y., Wang J. Fenerf: Face editing in neural radiance fields. Proceedings of the IEEE/CVF Conference on Computer Vision and Pattern Recognition.

[166] Wang S., Wang O., Owens A., Zhang R., Efros A. Detecting photoshopped faces by scripting photoshop. Proceedings of the IEEE/CVF International Conference on Computer Vision.

[167] Du CX T., Trung H.T., Tam P.M., Hung NQ V., Jo J. Efficient-Frequency: A hybrid visual forensic framework for facial forgery detection. Proceedings of the IEEE Symposium Series on Computational Intelligence (SSCI).

[168] Deepfake in the Wild Dataset. https://github.com/deepfakeinthewild/deepfake-in-the-wild.

[169] Rathgeb C., Nichols R., Ibsen M., Drozdowski P., Busch C. (2022). Busch. Crowd-powered Face Manipulation Detection: Fusing Human Examiner Decisions. arXiv.

[170] Phillips P., Wechsler H., Huang J., Rauss P.J. (1998). The FERET database and evaluation procedure for face-recognition algorithms. Image Vis. Comput..

[171] Guo Z., Yang G., Zhang D., Xia M. (2023). Rethinking gradient operator for exposing AI-enabled face forgeries. Expert Syst. Appl..

[172] Li Y., Chen X., Wu F., Zha Z.J. Linestofacephoto: Face photo generation from lines with conditional self-attention generative adversarial networks. Proceedings of the 27th ACM International Conference on Multimedia.

[173] Xia W., Yang Y., Xue J.H., Wu B. TediGAN: Text-Guided Diverse Face Image Generation and Manipulation. Proceedings of the IEEE/CVF Conference on Computer Vision and Pattern Recognition.

[174] Song H., Woo S., Lee J., Yang S., Cho H., Lee Y., Choi D., Kim K. Talking Face Generation with Multilingual TTS. Proceedings of the IEEE/CVF Conference on Computer Vision and Pattern Recognition.

[175] Zen H., Dang V., Clark R., Zhang Y., Weiss R.J., Jia Y., Chen Z., Wu Y. (2019). LibriTTS: A Corpus Derived from LibriSpeech for Text-to-Speech. Interspeech.

[176] Shi Y., Bu H., Xu X., Zhang S., Li M. (2021). AISHELL-3: A Multi-Speaker Mandarin TTS Corpus. Interspeech.

[177] Li Z., Min M., Li K., Xu C. StyleT2I: Toward Compositional and High-Fidelity Text-to-Image Synthesis. Proceedings of the IEEE/CVF Conference on Computer Vision and Pattern Recognition.

[178] Wang S., Wang O., Zhang R., Owens A., Efros A. CNN-generated images are surprisingly easy to spot… for now. Proceedings of the IEEE/CVF Conference on Computer Vision and Pattern Recognition.

[179] Pu J., Mangaokar N., Wang B., Reddy C., Viswanath B. Noisescope: Detecting deepfake images in a blind setting. Proceedings of the Annual Computer Security Applications Conference.

[180] Yousaf B., Usama M., Sultani W., Mahmood A., Qadir J. (2022). Fake visual content detection using two-stream convolutional neural networks. Neural Comput. Appl..

[181] Nowroozi E., Conti M., Mekdad Y. (2022). Detecting high-quality GAN-generated face images using neural networks. arXiv.

[182] Ferreira A., Nowroozi E., Barni M. (2021). VIPPrint: Validating Synthetic Image Detection and Source Linking Methods on a Large Scale Dataset of Printed Documents. J. Imaging.

[183] Boyd A., Tinsley P., Bowyer K., Czajka A. CYBORG: Blending Human Saliency Into the Loss Improves Deep Learning-Based Synthetic Face Detection. Proceedings of the IEEE/CVF Winter Conference on Applications of Computer Vision.

[184] Karras T., Aittala M., Hellsten J., Laine S., Lehtinen J., Aila T. (2020). Training generative adversarial networks with limited data. Adv. Neural Inf. Process. Syst..

[185] Karras T., Aittala M., Laine S., Härkönen E., Hellsten J., Lehtinen J., Aila T. (2021). Alias-free generative adversarial networks. Adv. Neural Inf. Process. Syst..

[186] Banerjee S., Bernhard J.S., Scheirer W.J., Bowyer K.W., Flynn P.J. SREFI: Synthesis of realistic example face images. Proceedings of the IEEE International Joint Conference on Biometrics.

[187] Mishra S., Shukla A.K., Muhuri P.K. (2022). Explainable Fuzzy AI Challenge 2022: Winner’s Approach to a Computationally Efficient and Explainable Solution. Axioms.

[188] Adadi A., Berrada M. (2018). Peeking Inside the Black-Box: A Survey on Explainable Artificial Intelligence (XAI). IEEE Access.

[189] Das A., Rad P. (2020). Opportunities and challenges in explainable artificial intelligence (xai): A survey. arXiv.

